# Pediatric Antifungal Prescribing Patterns Identify Significant Opportunities to Rationalize Antifungal Use in Children

**DOI:** 10.1097/INF.0000000000003402

**Published:** 2021-11-11

**Authors:** Laura Ferreras-Antolín, Adam Irwin, Ayad Atra, Faye Chapelle, Simon B. Drysdale, Marieke Emonts, Paddy McMaster, Stephane Paulus, Sanjay Patel, Menie Rompola, Stefania Vergnano, Elizabeth Whittaker, Adilia Warris

**Affiliations:** From the * Medical Research Council Centre for Medical Mycology, University of Exeter, United Kingdom; † St George’s University Hospitals NHS Foundation Trust, London, United Kingdom; ‡ Department of Paediatric Infectious Diseases, Great Ormond Street Hospital for Children, London, United Kingdom; § The University of Queensland Centre for Clinical Research, Brisbane, Australia; ¶ Department of Paediatric Oncology, Royal Marsden Hospital, Downs Road, Sutton, London, United Kingdom; ‖ Department of Infectious Diseases and Immunology, Evelina Children Hospital, London, United Kingdom; ** Oxford Vaccine Group, Department of Paediatrics, University of Oxford, Oxford, United Kingdom; †† Department of Paediatric Immunology, Infectious Diseases and Allergy, Great North Children’s Hospital, Newcastle upon Tyne Hospitals NHS Foundation Trust, Newcastle upon Tyne, United Kingdom; ‡‡ Institute of Cellular Medicine, Newcastle University, Newcastle upon Tyne, United Kingdom; §§ Department of Paediatric Infectious Diseases, Royal Manchester Childrens´ Hospital, Manchester, United Kingdom; ¶¶ Institute of Infection and Global Health, University of Liverpool, Liverpool, United Kingdom; ‖‖ Department of Paediatric Infectious Diseases, University Hospital Southampton NHS Foundation Trust, United Kingdom; *** Dept. of Paediatric Haematology and Oncology, Leeds General Infirmary, Leeds, United Kingdom; ††† Department of Paediatric Infectious Diseases, Bristol Royal Hospital for Children, Bristol, United Kingdom; ‡‡‡ Paediatric Infectious Diseases, Imperial College Healthcare NHS Trust and Section of Paediatrics, Department of Infectious Diseases, Imperial College, London, United Kingdom.

**Keywords:** antifungals, stewardship, prescriptions, children, amphotericin B, azoles

## Abstract

**Objective:**

The need for pediatric antifungal stewardship programs has been driven by an increasing consumption of antifungals for prophylactic and empirical use. Drivers and rational of antifungal prescribing need to be identified to optimize prescription behaviors.

**Methods:**

A prospective modified weekly Point Prevalence Survey capturing antifungal prescriptions for children (> 90 days to < 18 years of age) in 12 centers in England during 26 consecutive weeks was performed. Demographic, diagnostic and treatment information was collected for each patient. Data were entered into an online REDCap database.

**Results:**

One thousand two hundred fifty-eight prescriptions were included for 656 pediatric patients, 44.9% were girls, with a median age of 6.4 years (interquartile range, 2.5–11.3). Most common underlying condition was malignancy (55.5%). Four hundred nineteen (63.9%) received antifungals for prophylaxis, and 237 (36.1%) for treatment. Among patients receiving antifungal prophylaxis, 40.2% did not belong to a high-risk group. In those receiving antifungal treatment, 45.9%, 29.4%, 5.1% and 19.6% had a diagnosis of suspected, possible, probable of proven invasive fungal disease (IFD), respectively. Proven IFD was diagnosed in 78 patients, 84.6% (n = 66) suffered from invasive candidiasis and 15.4% (n = 12) from an invasive mold infection. Liposomal amphotericin B was the most commonly prescribed antifungal for both prophylaxis (36.6%) and empiric and preemptive treatment (47.9%). Throughout the duration of the study, 72 (11.0%) patients received combination antifungal therapy.

**Conclusions:**

Antifungal use in pediatric patients is dominated by liposomal amphotericin B and often without evidence for the presence of IFD. A significant proportion of prophylactic and empiric antifungal use was seen in pediatric patients not at high-risk for IFD.

The need for pediatric antifungal stewardship programs (pAFS) has been driven by an increasing consumption of antifungals for prophylactic and empirical use.^1–3^ Although partly explained by the challenges in the diagnosis of invasive fungal disease (IFD) and its associated high mortality, a poor understanding of who is at risk and timely access to diagnostic modalities is likely to contribute to an overuse of antifungals. It is recognized that some cohorts of pediatric patients are at high-risk of IFD,^4^ although new risk factors are being identified related to developments in immunosuppressive and immunomodulatory treatments.^5,6^ The current arsenal of fungal diagnostic tools has enabled a move away from an empiric to a preemptive management approach, which has been shown to be safe in reducing antifungal consumption.^7^ Overuse and inappropriate use of antifungals is expensive, drives antifungal resistance and is associated with increased adverse events.^8^ Antifungal stewardship (AFS) programs aim to reduce inappropriate antifungal use and to improve patient outcomes while reducing the evolution and spread of antifungal resistance. There is ample evidence from adult populations that AFS programs improve performance measures and decrease antifungal consumption.^9–11^ Currently, evidence on the value of pAFS programs is scarce.

The aims of the Paediatric Antifungal Stewardship: Optimising Antifungal Prescription in Children study are to obtain an in-depth insight into antifungal prescribing behaviors in pediatric patients and to identify the main gaps in knowledge with respect to antifungal prescribing.

## METHODS

### Study Design

A prospective modified weekly point prevalence survey (PPS) capturing antifungal prescriptions for children (> 90 days–< 18 years of age) in 12 hospitals in England during 26 consecutive weeks (June 2017 to January 2018) was performed. Each center collected the data on a specific day of each week.

### Study Definitions

A patient was defined at high-risk for IFD if they suffered from any of the following: acute myeloid leukemia, relapsed acute myeloid leukemia, relapsed acute lymphoid leukemia, severe aplastic anemia, chronic granulomatous disease, severe combined immunodeficiency or had received a hematopoietic stem cell transplant (HSCT).^12,13^ IFD was classified as possible, probable and proven based on the European Organization for Research and Treatment of Cancer/Mycoses Study Group criteria.^14^ Antifungal use included both prophylaxis and treatment. Treatment was defined as empiric (fever driven), preemptive (diagnostic driven) and targeted (evidence for IFD).^15^ The presence of the following risk factors were collected: central venous catheter, receipt of chemotherapy, corticosteroids > 0.3 mg/kg/d, other immunosuppressive therapy, persistent neutropenia (neutrophils < 0.5 × 10^9^/L for ≥ 10 days), use of broad-spectrum antibiotic (eg, piperacillin-tazobactam, meropenem, third- or fourth-generation cephalosporins, amoxicillin/clavulanic acid) for ≥ 5 days, parenteral nutrition, abdominal surgery, graft-versus-host disease, known *Candida* spp. colonization within 2 weeks before inclusion. Changes in dose, dosing regimen or route of administration were counted as separate prescriptions. The proportion of patients on antifungals per ward was calculated from the total of admissions at each individual ward during the successive PPS.

### Data Collection

Demographic, diagnostic and treatment information were collected for each patient. Changes in the certainty of the IFD diagnosis and the antifungal prescriptions were captured while the patient was hospitalized. Data were collected via REDCap (Research Electronic Data Capture, Vanderbilt University, Nashville, TN), a web-based application in which the investigators of the participating hospitals entered data online.

### Statistical Analysis

The quantitative variables were expressed as mean ± SD when they followed a normal distribution, or as median and interquartile range (IQR) when they had a non-normal distribution. Categorical variables were reported as frequency of distribution or rates and expressed as 2 × 2 tables. The distribution of factors by category of risk for IFD and the changes on treatment were analyzed using the χ^2^/Fisher tests or ANOVA test according to variable characteristics. Estimates were displayed using 95% confidence intervals. The statistical significance was defined as *P* < 0.05. All statistical analyses were performed using Stata SE software version 14.0.

## RESULTS

Nine hundred and nineteen children > 90 days old were included. Patients admitted to neonatal wards (n = 6) and those being prescribed oral nystatin only (n = 257), were excluded from the analysis. The remaining 656 patients were included. Each weekly PPS involved a mean of 100 ± 10 patients (SD 10). Each individual patient was included in the study for a median of one PPS week (IQR, 1–3). 9.8% (64/656) of the children were included in the PPS for ≥ 8 weeks, and 4.4% (29/656) ≥ 12 weeks.

### Demographics and Underlying Condition

Median age was 6.4 years (IQR, 2.5–11.3). The most common underlying condition was malignancy, n = 364 (55.5%). Seventy patients (10.7%) were diagnosed with a primary immunodeficiency (PID) and 209 (31.9%) had other underlying conditions or were previously healthy children (Table 1). Of the 656 children, 419 (63.9%) received antifungals for prophylaxis, and 237 (36.1%) for treatment at inclusion. Patients with PID and HSCT recipients received a higher proportion of antifungal prophylaxis compared with antifungal treatment (13.1% vs. 6.3% and 34.6% vs. 13.9%, respectively, *P* < 0.01) (Table 2).

**TABLE 1.** Characteristics of the Study Population

Patients (n)	656	
Age (y)	6.4 y (IQR, 2.5–11.3)	
Sex, female	293 (44.9%)	
Underlying conditions,* n (%)		
Malignancy	364 (55.5%)	
ALL		85 (23.5%)
Relapsed ALL		68 (18.8%)
AML		60 (16.6%)
Relapsed AML		16 (4.4%)
Other leukemias		25 (6.9%)
Non-Hodgkin lymphoma		22 (6.1%)
Hodgkin lymphoma		4 (1.1%)
Solid tumors		80 (20.2%)
Not specified		4 (1.1%)
Underlying condition PID	70 (10.7%)	
(S)CID		25 (35.7%)
Chronic granulomatous disease		12 (17.2%)
Chronic mucocutaneous candidiasis		1 (1.4%)
Other types of PID		32 (45.7%)
Underlying condition “other”	207 (31.6%)	
Hematologic disorder without bone marrow suppression		30 (14.6%)
Congenital heart disease (surgical)		24 (11.7%)
SOT		19 (9.2%)
CF		17 (8.3%)
Disorder with bone marrow suppression		14 (6.8%)
Gastrointestinal disease		14 (6.8%)
Surgical abnormality/condition		13 (6.3%)
None		12 (5.8%)
Post-HSCT		12 (5.8%)
Other†		52 (25.1%)
Risk factors, n (%)		
CVC	501 (76.4)	
Chemotherapy	328 (50.0)	
Broad-spectrum antibiotic (≥ 5 d)	267 (40.7)	
Peritransplant (HSCT or SOT)	197 (30.0)	
Prolonged neutropenia (≥ 10 d)	191 (29.1)	
Immunosuppressive therapy (nonchemotherapy)	174 (26.5)	
Steroid treatment (dose >0.3 mg/kg/d)	107 (16.3)	
Parenteral nutrition	89 (13.6)	
*Candida* colonization	68 (10.4)	
Abdominal surgery	52 (7.9)	
GvHD	38 (5.8)	

*No underlying condition reported in 15 patients.

†Categories with < 10 patients in each group, including inborn errors of metabolism; nonsurgical heart disease; chronic renal disease; chromosomal/single gene disorder; chronic neurologic condition; rheumatologic or inflammatory condition; neurosurgical abnormality; chronic respiratory disease; chronic endocrinologic disease.

(S)CID indicates severe combined immunodeficiency; ALL, acute lymphoblastic leukemia; AML, acute myeloid leukemia; CF, cystic fibrosis; CVC, central venous catheter; GvHD, graft-versus-host disease; ICU, intensive care unit; SOT, solid organ transplant.

**TABLE 2.** Rationale for Antifungal Prescription Grouped by Underlying Condition

Underlying condition,* N (%)	Prophylaxis, n = 419	Treatment, n = 237	*P* value	Total, n = 656
Malignancy	230 (54.8)	134 (56.5)	0.664	364 (55.5)
Leukemia	173 (75.2)	80 (59.8)		253 (71.4)
Lymphoma	15 (6.5)	11 (8.3)		26 (7.3)
Solid organ tumor	38 (16.5)	42 (31.2)		80 (22.6)
Other	4 (1.8)	1 (0.7)		5 (1.4)
HSCT recipients†	145 (34.7)	33 (13.9)	<0.01	178 (27.1)
Primary Immunodeficiency	55 (13.2)	15 (6.3)	<0.01	70 (10.7)
Others	126 (30.1)	81 (34.2)	0.286	207 (31.6)

*No underlying condition reported in 15 patients.

†Irrespective of underlying condition.

Most children receiving antifungals for therapeutic purposes had no diagnosis of IFD, 45.9% (95% CI, 41.5%–50.3%). Of those with a diagnosis of IFD, 29.4% (95% CI, 26.3%–32.5%) had a possible, 5.1% (95% CI, 3.4%–6.8%) probable and 19.6% (95% CI, 17.2%–21.9%) proven IFD. Among the children with a proven IFD for which microbiologic data was provided (n = 78), invasive candidiasis was diagnosed in 66 (84.6%), invasive aspergillosis in 5 (6.4%) and other invasive mold infections in 7 (9.0%). Of the patients who were started on antifungal treatment at inclusion (n = 237), only 25 (10.6%) had a change in the certainty of the IFD diagnosis within the successive weeks.

Most patients receiving antifungals had at least one risk factor for IFD (n = 629, 95.9%). A single risk factor was reported in 95 patients (14.5%), 2 in 136 (20.7%), and ≥ 3 in 398 (60.7%) (Table 1).

Among the patients receiving antifungal prophylaxis, 40.2% did not belong to a high-risk group, with 71.4% of the non-high-risk group having ≥ 2 risk factors. Among the non-high-risk patients receiving empiric treatment, 72.6% had ≥ 2 risk factors (Table 3).

**TABLE 3.** Distribution of Number of Risk Factors by Rationale of Treatment: Prophylaxis and Empiric Treatment

Number of risk factors, n (%)	Prophylaxis (n = 418)			Empiric treatment (n = 107)		
	High risk (n = 250, 59.8%)	Non-high risk (n = 168, 40.2%)	*P* value	High risk (n = 34, 31.8%)	Non-high risk (n = 73, 68.2%)	*P* value
None	5 (2)	16 (9.5)	<0.01	0 (0)	0 (0)	<0.01
1	22 (8.9)	32 (19.0)		1 (2.9)	20 (27.4)	
2	40 (16)	54 (32.1)		1 (2.9)	19 (26)	
≥ 3	183 (73.2)	66 (39.3)		32 (94.1)	34 (46.6)	

### Ward of Admission

The majority of the patients were admitted to hematology-oncology wards (n = 313; 47.7%), followed by HSCT wards (n = 130, 19.8%) and pediatric intensive care unit (PICU; n = 88, 13.4%). The proportion of admitted children receiving antifungals was highest on the HSCT wards: 59.8% (95% CI, 56.5%–63.2%), followed by hematology-oncology wards: 38.3% (95% CI, 36.3%–40.3%), and PICU: 17.6% (95% CI, 15.5%–19.7%).

### Antifungal Prescriptions

The total number of prescriptions was 1258. Most prescriptions were for prophylaxis, 70.7% (95% CI, 62%–79.5%), with 29.3% (95% CI, 20.5%–38%) for treatment. Among the different types of treatment, 29.9% (95% CI, 21.3%–38.5%) were for empiric treatment; 57.4% (95% CI, 46.2%–68.5%) for preemptive treatment and 12.8% (95% CI, 8.2%–17.3%) for targeted treatment.

Liposomal amphotericin B (L-AmB) was the most commonly prescribed antifungal (n = 474, 37.7%), followed by mold-active azoles (n = 459, 36.5%). Of the mold-active azoles, itraconazole was prescribed most commonly (n = 265, 49.2%) (Table 4).

**TABLE 4.** Rationale for Prescription of Specific Antifungal Agents

Agent, n (%)	Prophylaxis (n = 752)	Treatment			Total (n = 1227)*
		Empiric (n = 189)	Preemptive (n = 172)	Targeted (n = 114)	
L-AmB	275 (36.6)	89 (47.1)	84 (48.8)	19 (16.7)	467
Itraconazole	260 (34.6)	1 (0.5)	1 (0.6)	3 (2.6)	265
Voriconazole	48 (6.4)	18 (9.5)	30 (17.4)	14 (12.3)	110
Posaconazole	47 (6.2)	9 (4.8)	7 (4.1)	8 (7.0)	71
Isavuconazole	0	0	0	1 (0.9)	1
Fluconazole	85 (11.3)	46 (24.3)	13 (7.6)	49 (43.0)	193
Micafungin	26 (3.4)	11 (5.8)	28 (16.3)	3 (2.6)	68
Caspofungin	8 (1.1)	10 (5.3)	7 (4.1)	11 (9.6)	36
Anidulafungin	0	0	0	1 (0.9)	1
Flucytosine	0	0	0	5 (4.4)	5
Other AmB	3 (0.4)	5 (2.6)	2 (1.2)	0	10

*Total prescriptions 1258, from those, 1227 with full information on rational.

Other AmB: amphotericin B deoxycholate and lipids formulations of amphotericin B.

The 2 most common agents prescribed for prophylaxis were L-AmB (n = 275 prescriptions, 36.6%) and itraconazole (n = 260 prescriptions, 34.6%). Most of the L-AmB prescriptions for prophylaxis were in patients with an underlying malignancy [170/275 (61.8%)]. Of all the patients who were on L-AmB prophylaxis (n = 192) or itraconazole (n = 177), 66.1% (127/192) and 74.6% (132/177), respectively, were at high-risk for IFD. Prescriptions for fluconazole prophylaxis (n = 81) were mainly observed in non-high-risk patients (64/81; 79.0%), including those with non-high-risk malignancies (n = 23) and SOT recipients (n = 14).

L-AmB was the antifungal of choice for either empiric [89/189 (47.1%)] or preemptive treatment [84/172 (48.8%)]. Interestingly, the second most commonly prescribed antifungal agent for empiric treatment was fluconazole (46/189, 24.3%) to predominantly non-high-risk patients (42/46, 91.3%).

When comparing the total prescriptions for prophylaxis and empiric treatment (Table 4) with those prescribed to patients with underlying malignancies and HSCT only (Table 5), differential prescription behaviors were noted. The high-risk patients presented a higher proportion of itraconazole prophylaxis prescriptions compared with the total cohort (41.6% vs. 34.6%); whereas the proportion of fluconazole prophylaxis was lower (1.1% vs. 11.3%). Analysis of the prescriptions for empiric treatment showed a higher proportion of high-risk patients received L-AmB compared with the whole cohort (56.8% vs. 47.1%). Fluconazole was rarely prescribed as empiric treatment to high-risk patients (4.9%).

**TABLE 5.** Proportion of Prescriptions of the Different Agents as Prophylaxis or Empiric Treatment in the Groups With Underlying Malignancies and HSCT Categorized by Risk

Antifungal agent	Prophylaxis			Empiric treatment		
	High risk (n = 449)	Non-high risk + neutropenia (n = 54)	Solid tumors (n = 97)	High risk (n = 81)	Non-high risk + neutropenia (n = 33)	Solid tumors (n = 33)
L-AmB	175 (39.0%)	20 (37.0%)	42 (43.3%)	46 (56.8%)	17 (51.5%)	17 (51.5%)
Itraconazole	187 (41.6%)	12 (22.2%)	17 (17.5%)	1 (1.2%)	0	0
Voriconazole	26 (5.8%)	6 (11.1%)	15 (15.5%)	7 (8.6%)	3 (9.0%)	4 (12.2%)
Posaconazole	36 (8.0%)	3 (5.6%)	0	7 (8.6%)	1 (3.0%)	0
Fluconazole	5 (1.1%)	8 (14.8%)	15 (15.5%)	4 (4.9%)	8 (24.2%)	8 (24.2%)
Echinocandins	18 (4.0%)	5 (9.2%)	7 (7.2%)	12 (14.8%)	4 (12.1%)	3 (9.1%)
Other	2 (0.4%)	0	1 (1.0%)	4 (4.9%)	0	1 (3.0%)

High risk: post-HSCT patients, relapsed acute lymphoblastic leukemia, acute myeloid leukemia, relapsed acute myeloid leukemia, Hodgkin lymphoma. Non-high risk: acute lymphoblastic leukemia (regardless of chemotherapy protocol), other leukemias, non-Hodgkin lymphomas.

### Combination Therapy

At inclusion, 47 (7.2%) children received combination therapy with 2, and one child with 3 antifungals. Throughout the duration of the study, a total of 72 (11.0%) patients received dual, and 2 (0.3%) received triple antifungal therapy. Of those on combination therapy, 58 (78.4%) were patients at high-risk for IFD. Combination therapy accounted for 108 prescriptions, of which 33 (30.9%) were prescriptions for proven or probable IFD, whereas 31 (29.0%) and 44 (40.2%) were in patients with possible and no diagnosis of IFD.

### Prescription Changes

**FIGURE 1.**
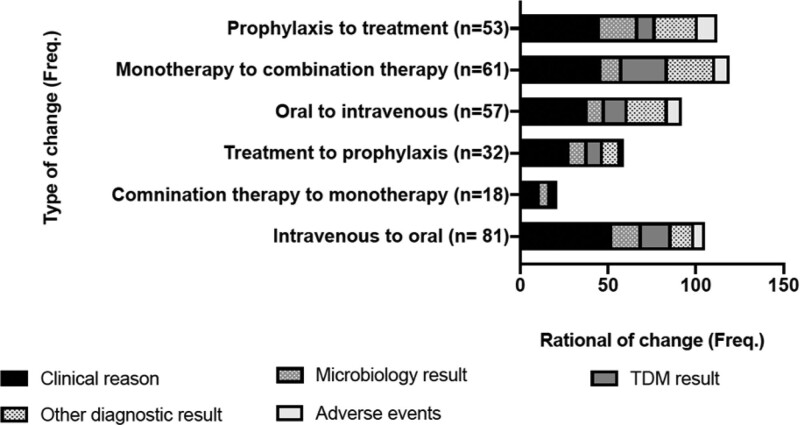
Frequency of prescription changes along the successive PPS weeks by type of change and rational registered for this change. Numbers within brackets represent number of patients. TDM indicates therapeutic drug monitoring.

During the successive PPS weeks, 509 changes were made to prescriptions affecting 259 (39.5%) patients. Of those, 311 (61.1%) changes were deescalation measures, either a switch from intravenous to oral or from combination therapy to monotherapy. A total of 155 (30.4%) were related to a change from treatment to prophylaxis (stepping-down). The rational for those changes were either based on clinical judgment (75.6% in deescalation and 91.6% for stepping-down). Figure 1 shows the differentiated rational for the prescription changes made.

## DISCUSSION

The results of this PPS show that the majority of pediatric antifungal prescriptions are for patients without evidence of IFD and that L-AmB is the most commonly used antifungal for both prophylactic and therapeutic purposes. A significant proportion of antifungal consumption was observed in pediatric patients whose underlying condition is not recognized as rendering them at high-risk for IFD.

The majority of the prescriptions (70%) were for antifungal prophylaxis, which is a higher figure than previously reported in a comparable, but single PPS, showing 46% of the antifungal prescriptions to be for antifungal prophylaxis.^1^ This higher proportion seems not to be due to a larger population of children being at risk, as in our study, 40% could not be categorized as being at high-risk to develop IFD. The practice of defining categories of pediatric patients being at low, moderate and high risk for IFD has been used to establish management recommendations for children with hematologic malignancies and those undergoing HSCT.^12^ For children with PID, defects in specific innate immune pathway (eg, STAT-3 deficiency), severe impairment of neutrophil function (chronic granulomatous disease) or T-cell function (severe combined immunodeficiency) are associated with a high-risk of developing IFD.^4,16^ For those high-risk pediatric patients, antifungal prophylaxis is recommended as antifungal prophylaxis has shown to decrease the incidence of IFD.^12,13,17^ Several studies have defined individual risk factors associated with the development of IFD. Prolonged neutropenia, the use of high-dose steroids, the presence of graft-versus-host disease, delayed lymphocyte engraftment are risk factors associated with the development of IFD in patients with hemato-oncologic disorders and HSCT recipients.^18,19^ Central venous catheters, parenteral nutrition, the prolonged use of vancomycin or agents with activity against anaerobic organisms enhances specifically the risk of invasive candidiasis in the intensive care setting, next to those admitted to hemato-oncology wards and HSCT units.^19–21^ Our results reflect this specified risk to a certain extent, as fluconazole was prescribed more commonly as a prophylactic agent outside the patient population being at high risk for IFD caused by both yeasts and molds. The combination of the underlying condition and the presence of risk factors are often taken into account when prescribing antifungal prophylaxis and empiric antifungal therapy. A number of predictive risk models for invasive candidiasis have been developed to guide antifungal therapy for children admitted to the PICU.^22–24^ Unfortunately, those models have either not been validated^22,23^ or failed to do so.^25^ Recently, a predictive risk model was described to estimate the 60-day probability of developing probable or proven invasive mold disease in hematology patients.^26^ Potential use of such prediction models is to allow for targeted biomarker screening and fine-tuning of management strategies (eg, prophylaxis, empiric therapy) thereby optimizing antifungal prescribing. Our study showed that amongst the non-high-risk population who were on either antifungal prophylaxis or received empirical treatment, less than half of the patients had three or more risk factors. Targeting the high number of antifungal prescriptions in non-high-risk pediatric populations is needed and will be of value to improve the rational use of antifungals.

L-AmB was the most frequently prescribed antifungal for prophylaxis. This is surprising as it is not licensed for prophylaxis, efficacy data are not known, and no dosing recommendations for prophylactic use exists. Experiences with L-AmB prophylaxis are restricted to single-center reports.^27,28^ The compatibility with vincristine, fewer drug-drug interactions and lack of the need to perform therapeutic drug monitoring, are the most likely reasons to prescribe L-AmB instead of a mold-active azole. Some clinical management guidelines mention L-AmB as an alternative antifungal prophylaxis for pediatric patients, although with a low evidence grading due to lack of data.^12,13^

The European Organization for Research and Treatment of Cancer/Mycoses Study Group Consensus Definitions provide a tool to make a diagnosis of IFD with a specified level of certainty.^14^ It has recently undergone a second revision and the revised version captures for the first time pediatric-specific signs and symptoms.^14^ It should be stressed, though, that these definitions are specifically intended for the purpose of facilitating epidemiologic and clinical research and do not aim to direct patient care. Our results might indirectly demonstrate that the host factors summarized in the Consensus Definitions are being used in the decision to prescribe antifungal therapy.

L-AmB was shown to be the antifungal of choice in almost 50% of patients for empirical and preemptive therapy. Both caspofungin and L-AmB are recommended for empirical treatment in hemato-oncologic patients with febrile neutropenia.^12,13,29^ From an AFS perspective, it is critical to review empiric antifungal therapy at least weekly, as most patients on empiric therapy will not have an IFD. Preemptive antifungal therapy is a diagnostic-driven approach enabling a more targeted antifungal therapy. Our data does not reflect this differentiation in approach based on the specific antifungal prescribed and may have several explanations. Current fungal biomarkers are only available for a restricted number of fungi (eg, *Candida* and *Aspergillus* species), abnormalities on CT-chest do not identify the causative fungus, and azole-resistant aspergillosis drives the use of L-AmB if cultures are negative and susceptibility testing is not performed. Fung et al^7^ published a meta-analysis comparing empirical and preemptive antifungal therapy in adult patients with high-risk febrile neutropenia and reported decreased antifungal use without increasing mortality and no major economic impact when the latter strategy was used. A Cochrane review published in 2015 reported insufficient data to support a preemptive approach in children,^30^ and this still remains an important research gap. In the meantime, 2 single-center studies have been published supporting the use of a preemptive approach in children with febrile neutropenia.^31,32^

The finding of a majority of the antifungal prescriptions for pediatric patients without evidence of IFD might indirectly reflect a limitation to access available fungal diagnostics tools. We have previously reported that the use of fungal diagnostic tools varied with a prolonged turn-around time being an important barrier for many centers.^33^ To improve antifungal prescribing in situations where IFD is clinically suspected, efforts should be focused on the access to timely fungal diagnostics. The current indirect fungal markers (eg, β-D-glucan, galactomannan, lateral flow assay) and polymerase chain reaction-based tests are of high value to exclude the presence of IFD and are able to guide decisions to rationalize antifungal use.^34^

It is worth to consider the limitations of our modified PPS. While changes in diagnosis of IFD and prescriptions were captured, qualitative information such as changes in the clinical condition of the patient and diagnostics results were not collected. Prescription data was only collected when patients were admitted to the hospital, and therefore, information on the duration of antifungal prophylaxis or treatment is lacking. In addition, the impact of local guidelines was not assessed.

The Paediatric Antifungal Stewardship: Optimising Antifungal Prescription in Children modified PPS has identified key areas to be addressed in a pAFS program to optimize antifungal prescribing. The unexplained high use of antifungal prophylaxis outside the defined high-risk populations, and a significant use of antifungals as treatment in patients with suspected IFD. It reflects the challenge of recognizing who is at high risk, the difficulties encountered in either ruling in or ruling out of the presence of IFD with the current diagnostic tools, as well as timely access to fungal diagnostic tools. Further development and validation of predictive risk models in specific patient populations has the potential to more precisely define the patient at risk and to optimize antifungal use. Providing targeted educational programs and improving the access to fungal diagnostic and reporting of results are urgently needed. Establishing pAFS teams within individual centers is most likely to be of important value to further improve rational antifungal prescription behavior as a recent single pediatric center study has shown.^35^

## ACKNOWLEDGMENTS

We are grateful for the support received at each site in the data collection and want to thank the following colleagues: John Booth (Great Ormond Street Hospital for Children, London); Lucia Van Bruggen (Royal Marsden Hospital, Sutton, London); Alicia Demirjian (Evelina Children Hospital, London); Jessy-Anne Hudson and Robin Basu Roy (Department of Paediatrics, University of Oxford, Oxford); Helen Young and Layla Campbell (Great North Children’s Hospital, Newcastle upon Tyne Hospitals NHS Foundation Trust, Newcastle upon Tyne); Selma Eldirdiry Mohamed (Royal Manchester Childrens’ Hospital, Manchester); Claire Crouch and Ruth Cantwell (Alder Hey Children’s NHS Foundation Trust, Liverpool); Jessica Head (University Hospital Southampton NHS Foundation Trust, Southampton); Sally Kinsey and Alexandra Lu (Leeds General Infirmary, Leeds); and Emily Chesshyre (Bristol Royal Hospital for Children, Bristol) and Lucia Zombori (Imperial College Healthcare NHS Trust, London).
